# Supplementary material for: Doubling Power Output of Starch Biobattery Treated by the Most Thermostable Isoamylase from an Archaeon Sulfolobus tokodaii
Source: Sci Rep. 2015 Aug 20;5:13184. doi: 10.1038/srep13184 (PMC4542469; doi:10.1038/srep13184)
Supplement: Supplementary Information [file srep13184-s1.doc]

**Doubling Power Output of Starch Biobattery Treated by the Most Thermostable Isoamylase from an Archaeon *Sulfolobus tokodaii***

**Kun Cheng,^1,2^ Fei Zhang,^3^ Fangfang Sun,^3^ Hongge Chen,^1*^ Y-H Percival Zhang^2,3,4*^**

^1^ College of Life Sciences, Henan Agricultural University, 95 Wenhua Road, Zhengzhou, 450002, China

^2^ Biological Systems Engineering Department, Virginia Tech, 304 Seitz Hall, Blacksburg, Virginia 24061, USA

^3^ Cell Free Bioinnovations Inc. 1800 Kraft Drive, Suite 222, Blacksburg, Virginia 24060, USA

^4^ Tianjin Institute of Industrial Biotechnology, Chinese Academy of Sciences, 32 West 7^th^ Avenue, Tianjin Airport Economic Area, Tianjin 300308, China

**^*^**Corresponding authors’ E-mails:

HC: [honggeyz@163.com](mailto:honggeyz@163.com)

YPZ: [ypzhang@vt.edu](mailto:ypzhang@vt.edu)

**Revised MS Submitted to Scientific Reports**

# **Abstract**

Biobattery, a kind of enzymatic fuel cells, can convert organic compounds (e.g., glucose, starch) to electricity in a closed system without moving parts. Inspired by natural starch metabolism catalyzed by starch phosphorylase, isoamylase is essential to debranch alpha-1,6-glycosidic bonds of starch, yielding linear amylodextrin – the best fuel for sugar-powered biobattery. However, there is no thermostable isoamylase stable enough for simultaneous starch gelatinization and enzymatic hydrolysis, different from the case of thermostable alpha-amylase. A putative isoamylase gene was mined from megagenomic database. The open reading frame ST0928 from a hyperthermophilic archaeron *Sulfolobus tokodaii* was cloned and expressed in *E. coli*. The recombinant protein was easily purified by heat precipitation at 80^o^C for 30 min. This enzyme was characterized and required Mg^2+^ as an activator. This enzyme was the most stable isoamylase reported with a half lifetime of 200 min at 90^o^C in the presence of 0.5 mM MgCl_2_, suitable for simultaneous starch gelatinization and isoamylase hydrolysis. The cuvett-based air-breathing biobattery powered by isoamylase-treated starch exhibited nearly doubled power outputs than that powered by the same concentration starch solution, suggesting more glucose 1-phosphate generated.

**Keywords**: Bioelectricity, Enzymatic Fuel Cells, Isoamylase, Starch Hydrolysis, Sugar Biobattery

# **Introduction**

Biological fuel cells are emerging electro-biochemical devices that directly convert chemical energy from a variety of fuels into electricity by using low-cost biocatalysts enzymes or microorganisms instead of costly precious metals [^1-3^](#_ENREF_1). Compared to microbial fuel cells, enzymatic fuel cells usually generate much higher power densities in terms of mW/cm^2^ , suggesting their great potential for powering a variety of portable electronic devices . Inspired by the metabolism of living organisms that can utilize complex organic compounds (e.g., starch, glycogen) as stored energy sources and release glucose 1-phosphate slowly for catabolism, polysaccharide-powered enzymatic fuel cells may be more promising than mono-saccharide powered enzymatic fuel cells [^2^](#_ENREF_2) because polysaccharide has 11% higher energy density than glucose, has a much lower osmotic pressure than glucose and release chemical energy stepwise. A recent breakthrough of complete oxidation of glucose units of maltodextrin based on an ATP-free synthetic enzymatic pathway lead to a high-energy density biobattery [^2^](#_ENREF_2). But alpha-1,4,6-D-glucose branch-points in amylopectin, a dominant component of plant starch, and maltodextrin (**Fig. 1a**) cannot be converted to glucose 1-phosphate catalyzed by alpha-glucan (starch) phosphorylase, resulting in a waste of the fuel and decreased energy density.

Isoamylase (IA, EC 3.2.1.68) hydrolyzes alpha-1,6-glucosidic branch linkages in glycogen and amylopectin (**Fig. 1a**). The enzyme is able to hydrolyze both inner and outer branching points of amylopectin, and is commonly used in combination with other enzymes, such as alpha-amylase, beta-amylase, and glucoamylase to produce maltose and glucose from starch. In contrast, another commonly-used de-branching enzyme pullulanase (EC 3.2.1.41) prefer hydrolyzing very short branched dextrin that is remaining oligosaccharides of enzymatic hydrolysis of amylopectin catalyzed by alpha-amylase and/or beta-amylase [^6^](#_ENREF_6). Therefore, pullulanase is an important enzyme, along with alpha-amylase, glucoamylase, and beta-amylase, used for the production of glucose from starch. In terms of glucose 1-phosphate generation, isoamylase is very important to convert amylopectin to linear amylodextrin with a degree of polymerization of 20-30 from amylopectin. However, few thermostable or thermotolerant isoamylases were studied compared to thermostable alpha-amylase used in the starch industry. None of them are stable enough for simultaneous starch gelatinization and enzymatic hydrolysis. Pre-mixing of starch granules with thermostable isoamylase is very important to decrease mixing energy consumption for the high-viscosity starch slurry and generate the relatively homogeneous hydrolytic product -- amylodextrin.

Linear amylodextrin made from branched amylopectin catalyzed by isoamylase is different from maltodextrin made by alpha-amylase, which contains some branched points. In the purpose of ATP-free production of glucose 1-phosphate catalyzed by starch phosphorylase, amylopectin is better than maltodextrin for better glucose utilization efficiency and high weight slurry achieved. Such low-cost glucose 1-phosphate produced from starch and phosphate ions can be used to generate bioelectricity here, generate low-cost green hydrogen in distributed bioreactors , provide energy for cell-free protein synthesis , synthesize fine chemicals (e.g., 6-phophogluconate) [^13^](#_ENREF_13), and so on. Therefore, the production of amylodextrin or its short products (e.g., maltose) from starch by using isoamylase is becoming more and more important [^8^](#_ENREF_8).

Enzyme-based biocatalysis has become an attractive alternative to chemical catalysis because of its higher reaction selectivity and more modest reaction conditions . But most enzymes are not suitable for industrial applications due to their relatively poor stability. Discovery and utilization of thermoenzymes from hyperthermophilic microorganisms and exploding megagenome database is of great interest for numerous industrial applications [^15^](#_ENREF_15). *Sulfolobus tokodaii* was originally discovered in an acidic spa in Beppu Hot Springs of Kyushu Island, Japan, in the early 1980s [^16^](#_ENREF_16). It is a hyperthermophilic archeaon with an optimal growth temperature of 80 ^o^C and an optimal pH of 2.5-3.0. *S. tokodaii* strain 7 is the most investigated because it is the most abundant, can be easily isolated and cultivated in labs. Its genomic DNA sequence has been completed in 2001 [^16^](#_ENREF_16). *S. tokodaii* may be an invaluable source of intrinsically thermostable enzymes.

In this study, the open reading frame (ORF) ST0928 which was hypothesized to encode a glycoside hydrolase -- glycogen debranching enzyme (E.C.3.2.1.-) was cloned in *E. coli*. The recombinant enzyme was purified and characterized for the first time. Isoamylase-treated starch was tested to power biobattery compared to non-treated starch (**Fig. 1b**).

# **Results**

**Identification of a putative IA.**

Compared to thermostable alpha-amylase, isoamylase received less attention because its hydrolytic product – amylodextrin has limited applications. Approximately 10 isoamylases have been purified and characterized (**Table 1**). Among them, one from a hyperthemophilic archaeon *S. solfataricus* is thermostable [^7^](#_ENREF_7) and the other from *Bacillus lentus* is thermotolerant . But their lifetime at 90^o^C, a temperature needed for starch gelatinization, is not long enough (e.g., several hours for alpha-amylase) for simultaneous starch gelatinization and enzymatic hydrolysis.

We searched potential thermostable isoamylase genes by following the below protocol. First, we collected all characterized isoamylase protein sequences. Second, we blasted the known isoamylase protein sequences against the whole gene database of the National Center for Biotechnology Information (NCBI) and especially against special hypthermophilic micro-organisms, whose optimal growth temperature is more than 80^o^C. Third, we double checked possible thermostable isoamylase annotations in two other database -- the Kyoto Encyclopedia of Genes and Genomes (KEGG) and the glycoside hydrolase family 13 of CAZy (<http://www.cazy.org/>). It was found that an ORF (ST0928) was annotated to encode a putative glycogen debranching enzyme [^16^](#_ENREF_16). Its deduced amino acid sequence contains 716 amino acids and has a calculated molecular weight of 83.1 kDa. This predicted mature enzyme has a family 48 carbohydrate-binding module (17-108 AA) and a catalytic domain of alpha-amylase (204-545 AA) followed by an unknown function polypeptide (546-716 AA). This putative IA shared 80% and 79% identities with a well-characterized IA from the archaeon *S. solfataricus* [^7^](#_ENREF_7) and another putative IA from *S. acidocaldarius*, respectively, and much lower identities with reported bacterial IAs, such as *E. coli* (43%) [^18^](#_ENREF_18), *Archorbacter* sp. (53%) [^19^](#_ENREF_19), *Flavobacterium odoratum* [^20^](#_ENREF_20), *Pseudomonas amyloderamosa* (34%) [^21^](#_ENREF_21), [*Erwinia chrysanthemi*](javascript:Org('556')) (41%) [^22^](#_ENREF_22), *Bacillus* spp. (26%) , as well as one IA from plant *Phaseolus vulgaris* (43%) [^24^](#_ENREF_24). According to CAZy (http://www.cazy.org/), this putative IA belongs to glycoside hydrolase family 13, which includes more than 20 different kinds of hydrolases, such as alpha-amylase, pullulanase, cyclomaltodextrin glucanotransferase, isoamylase, trehaloe synthase, sucrose phosphorylase, and so on.

**Fig. 2** shows the three highly conserved amino acid sequences located in the catalytic domains among archaeal, bacterial and plant isoamylases. The three essential amino acid sites of this enzyme were Asp in region I, Glu in region II, and Asp in region III, in an agreement with

Asp375, Glu435, and Asp510 of the P. amyloderamosa isoamylase, all of which play a catalytic role in activities of the α-amylase family [^21^](#_ENREF_21).

**Expression and purification of isoamylase**

The ST0928 was sub-cloned into the T7-promoter plasmid pET20b by restriction enzyme-free, ligase-free Simple Cloning technique [^25^](#_ENREF_25). Two *E. coli* strains BL21(DE3) and Rosetta (DE3) were tested to express the recombinant IA with a His tag on its C terminus. Apparently, *E. coli* Rosetta was a better host than BL21 to express the soluble targeted enzyme (Fig. 3A, the left gel) because this gene contained a lot of rare codons in *E. coli*, including one three-rare codon cluster and several two-rare codon clusters. Although the host Rosetta can co-express the tRNAs for rare codons, a clear band with a molecular weight of ~81 kDa was observed in the pellet fraction by SDS-PAGE (**Fig. 3a**, Lane P), suggesting a significant amount of inclusion body formed. The His-tag enzyme was purified by affinity adsorption on nickel-charged resins. Alternatively, the cell lysate containing this enzyme was treated at 80^o^C for 30 min, to denature *E. coli* cellular proteins. After centrifugation, the targeted protein was the predominant band in the supernatant, being approximately 85% purity (Fig. **3a**, Lane HT). The protein recovery efficiency for nickel resin adsorption and heat precipitation were 81% and 98%, respectively. Approximately 10 mg of the purified His-tagged enzyme was purified from 200 mL of the cell culture grown in the LB media. This His-tagged enzyme had a specific activity of 6.4 IU/mg on amylopectin at 80^o^C based on the reducing ends generated. The specific activity of heat precipitated enzyme was approximately 89% of that purified from nickel resin adsorption, in consistent of SDS-PAGE data. Heat precipitation is the easiest approach for purifying relatively pure thermostable enzymes suitable for in vitro biocatalysis .

**Basic enzyme properties**

The optimal pH of this enzyme was tested in two buffers – acetate and phosphate on amylopectin (**Fig. 3b**). This enzyme had a narrow optimal pH 5.5 in the acetate buffers but a relatively broad pH range in the phosphate buffers. In 40 mM acetate buffer (pH 5.5), this enzyme exhibited the optimal temperature of 85^o^C and remained approximately 50% activity at 50^o^C (**Fig. 3c**), suggesting that this enzyme had a broad temperature range. The effects of the addition of 0.5 or 5 mM metal ions (i.e., CuCl_2_, FeCl_3_, ZnCl_2_, CaCl_2_, MgCl_2_, CoCl_2_, NiCl_2_, MnCl_2_) and EDTA on enzyme activities were studied in the acetate buffer (pH 5.5) at 80^o^C. The addition of EDTA regardless of its concentration caused protein aggregation and drastically decreased this enzyme activities, suggesting that some metal ions were important. Both MgCl_2_ and CaCl_2_ (0.5 or 5 mM) increased this enzyme activity, while 5 mM CoCl_2_, NiCl_2_, MnCl_2_ significantly decreased the enzyme activity; CuCl_2_, ZnCl_2_ and FeCl_3_ completely inhibited this enzyme activity.

Amylopectin was hydrolyzed by this enzyme under its optimal condition (e.g., acetate buffer (pH 5.5) containing 5 mM MgCl_2_ and 80^o^C) (**Fig. 4**). The branched amylopectin shows a typical brown-blue color after the iodine dying (**Fig. 4a**) because branched amylopectin cannot form coils and thus it does not form a complex with iodine. After this enzyme treatment, the solution turned a purple color (**Fig. 4a**), suggesting that linear amylodextrin forms a representative starch/iodine color – purple/deep blue. **Fig. 4b** shows the changes in absorption spectra of the iodine-staining solution for the amylopectin before and after the treatment of this enzyme. The absorbance increased and the maximum wavelength of absorption shifted to a longer wavelength from 530 to 560 nm. These results suggest that the enzyme hydrolyzed the 1,6-alpha-glycosidic linkage of branched amylopectin. This enzyme exhibited a very low activity on amylose (~5%) relative to that on amylopectin, indicating that this enzyme preferred hydrolyzing alpha-1,6-glycosidic bonds. This very low activity on amylose could be due to the high-sensitivity reducing end assay based on the BCA assay instead of the commonly-used Somogyi assay and/or some minor branches in natural amylose. Also, this enzyme can generate new reducing ends on long-chain maltodextrin (DE 4.0-7.0) but no new ends generated on short-chain maltodextrin (DE 16.5-19.5), suggesting that it cannot hydrolyze alpha-1,4,6-D-glucose branch-points for short maltodextrin, different from pullulanase. The above results seemed appropriate to refer to this enzyme as an isoamylase but its weak alpha-1,4-hydrolytic activity was not eliminated completely. This enzyme had a specific activity of 6.4 IU/mg on amylopectin at 80^o^C based on the reducing ends generated.

This isoamylase in the acetate buffer (pH 5.5) were very stable at temperatures of 60-80^o^C, less than 1% activity losses for 1 h, and remained 87% activity after 1 h incubation of 90^o^C. Surprisingly, this enzyme was more stable in the presence of 5 MgCl_2_ than the absence of bivalent ions (**Fig. 5**). The addition of MgCl_2_ resulted in a half lifetime of 200 min at 90^o^C. In contrast, CaCl_2_ decreased this enzyme stability greatly, resulting in a half lifetime of 35 min.

A de novo synthetic enzymatic pathway was designed to generate electricity from starch (**Fig. 1b)**. In it, alpha-glucan phosphorylase (αGP) cleaves alpha-1,4-glycosidic bonds from nonreducing ends of starch, maltodextrin or amylodextrin in the presence of phosphate, yielding glucose 1-phosphate; phosphoglucomutase (PGM) converts glucose 6-phosphate; glucose 6-phosphate dehydrogenase generates NADH from glucose 6-phosphate and release 6-phosphogluconate; diaphorase transfers hydrides from NADH via a mediator AQDS to anode. This pathway was slightly different from the previous pathway used [^28^](#_ENREF_28): (i) amylopectin instead of maltodextrin as the substrate, and (ii) non-immobilized AQDS instead of immobilized VK3 as the mediator. The entire sugar biobattery based on a typical plastic cuvette without mobile parts is shown in **Fig. 6d. Fig. 6** shows the results of electrochemical tests of sugar batteries powered by starch and isoamylase-treated starch. **Figs. 6a&b** display the polarization curves using isoamylase-treated or nontreated starch as the sugar biobattery’s substrate, respectively. When nontreated starch was used, the polarization curve shows that the open circuit potential (OCV) was 0.23 V with short connection current of 0.029 mA. At 0.14 V, the power density reached to a peak of 2.2 μW/cm^2^. In contrast, feeding the biobattery with isoamylase-treated starch, the maximum power density was almost doubled from 2.2 to 4.1 μW/cm^2^. In the meantime, short connection current increased to 0.042 mA, and OCV increased to 0.31 V. To eliminate the different cathode performance, individual potentials were recorded (data was not shown). Both of the cathode potentials were 0.53 V with different substrates. Only the anode leaded to varied whole cell performance, suggesting more glucose 1-phosphate generated from isoamylase-treated starch. To further confirm this testing result, cyclic voltammetry were recorded in two types of anolyte solutions. As shown in Fig. 6c, both of the starches showed very slight oxidation peaks which may result from low concentration starch (0.012% wt/v). Both oxidation peaks of isoamylase-treated and nontreated starch were approximately -300 mV relative to Ag/AgCl, but isoamylase-treated starch had higher current indicating isoamylase-treated starch was better than nontreated starch in the anode reaction.

# **Discussion**

Starch is the most widely used energy storage compound in nature. The catabolism of starch mediated by starch phosphorylase lead to a slow and nearly constant release of chemical energy (i.e., glucose 1-phosphate) in living cells that is different from that of the monomer glucose [^29^](#_ENREF_29). Amylodextrin made by isoamylase is much better than maltodextrin, a partially hydrolyzed starch fragment by alpha-amylase, because maltodextrin contains some 1,4,6 branched points, resulting in low glucose utilization efficiency. On the other side, amylopectin is a superior fuel to glucose because it has 11% higher energy density than glucose. An equivalent weight of amylopectin has a much lower osmotic pressure than glucose. Moreover, slowly-metabolized glucose 1-phosphate can provide more stable electricity generation in closed biobattery [^2^](#_ENREF_2).

This enzyme has the highest lifetime among all characterized isoamylases (Table 1). Due to its highest stability (i.e., a half lifetime of 200 min at 90^o^C), this hyper-thermophilic enzyme can be used in simultaneous starch gelatinization and enzymatic hydrolysis at ~90^o^C for several hours, like the case of alpha-amylase. This enzyme exhibited much better stability than the reported *S. solfataricus* IA, where the His-tag enzyme nearly lost all its activity after 120 min at 90^o^C [^7^](#_ENREF_7).

This enzyme has different metal ion requirement from other reported isoamylases. Compared its closest IA from *S. solfataricus*, which did not require any metal ions [^7^](#_ENREF_7), this enzyme required Mg^2+^ or Ca^2+^ for its maximum activity. Furthermore, its thermostability was improved greatly in the presence of Mg^2+^. This metal preference of this enzyme was a little different from those of *B. lentus* IA that preferred Ca^2+^ but Mg^2+^ was an inhibitor [^8^](#_ENREF_8).

In conclusion, this enzyme was the most stable isoamylase reported and had a half lifetime of 200 min at 90^o^C. Different from the closest IA from *S.* *solfataricus*, this required Mg^2+^ as an activator while EDTA impaired its activity greatly. Due to its highest stability, this enzyme can be used for simultaneous starch gelatinization and isoamylase hydrolysis, producing linear amylodextrin. Isoamylase-treated starch produced nearly doubled power outputs in a sugar biobattery relative to that powered by the same concentration starch.

# **Methods**

## **Chemical and strains.**

All chemicals were reagent grade, purchased from Sigma-Aldrich (St. Louis, MO, USA) and Fischer Scientific (Pittsburg, PA, USA), unless otherwise noted. Amylopectin from maize and maltodextrins (dextrose equivalent: 4.0-7.0, 13.0-17.0, and16.5-19.5) were purchased from Sigma-Aldrich. Pullulan was purchased from Aladdin (Fengxian, Shanghai, China). The DNA polymerase used was Phusion high-fidelity DNA polymerase from New England Biolabs (Ipswich, MA, USA). The protein marker (7-175kDa) was purchased from New England Biolabs (Ipswich, MA, USA). Primers were purchased from IDT (Coraville, IA). The PCR thermocycler was Eppendorf temperature gradient Mastercycler (Hauppauge, NY, USA). *S. tokodaii* strain 7 genomic DNA was purchased from DSMZ (Braunschweig, Germany). *E. coli* DH5α was used for DNA manipulation; *E. coli* BL21(DE3) and Rosetta (DE3) (Invitrogen, Carlsbad, CA, USA) and pET20b (+) (Novagen, Germany) were used for gene expression. *E. coli* strains were cultivated in the Luria–Bertani (LB) medium at 37°C. Ampicillin at 100 μg/mL was added in the *E. coli* medium.

## **Plasmid construction.**

The DNA fragment containing the ORF ST0928 was amplified by PCR with a pair of primers IF (5' AC TTTAA GAAGG AGATA TACAT atggt ttttt cacac aagga tagac cat 3') and IR (5' T CAGTG GTGGT GGTGG TGGTG atatt caatc ctcct atata ccatt gcgg 3') based on the genomic *S. tokodaii* DNA. A linear vector backbone was amplified based on pET20b (+) with a pair of primers VF (5' ccgc aatgg tatat aggag gattg aatat CACCA CCACC ACCAC CACTG A-3') and the reverse primer VR (5’ atg gtcta tcctt gtgtg aaaaa accat ATGTA TATCT CCTTC TTAAA GT 3’). The lower cases of primers matched the DNA sequences of the inserted gene and upper cases of primers matched the DNA sequences of the plasmid. The two PCR products were assembled by prolonged overlap extension PCR (POE-PCR) [^25^](#_ENREF_25). POR-PCR conditions were as followings: initial denaturation (30 s at 98°C), 25 cycles of denaturation (10 s at 98°C, annealing 10 s at 60°C, and elongation 72°C a rate of 2 kb/min), and a final extension step (10 min at 72°C). The POE-PCR product was transferred to *E. coli* DH5α, yielding plasmid pET20b-StIA.

## **Expression and purification of recombinant proteins**

Plasmid pET20b-StIA was transferred into *E. coli* Rosetta (DE3) or BL21(DE3). The *E. coli* culture was grown at 37°C in 250-mL Erlenmeyer flasks containing 50 mL of the LB medium. When the absorbance (A_600_) of the culture reached ca. 0.6, isoamylase expression was induced with 0.05 mM isopropyl-β-D-thiogalactoside for 16 h at 16 °C. The cells were harvested by centrifugation at 4 °C and washed twice with 20 mM of phosphate buffer (pH 7.4) containing 0.3 M NaCl. The cell pellets were suspended in the same buffer and lysed by ultra-sonication in an ice bath by the Brason disruptor model 450 under conditions (i.e., 3 s pulse on and 4 s off, total 300 s at 50% amplitude). After centrifugation, the soluble His-tagged isoamylase in the supernatant was purified using a packed column of Ni-charged resin (Bio-Rad, Profinity IMAC Ni-Charged Resin). The other *E. coli* cellular proteins were washed away with a binding buffer (20 mM PBS buffer (pH 7.4) containing 0.3 M NaCl and 10 mM imidazole). The adsorbed isoamylase was eluted with 20 mM PBS (pH 7.4) buffer containing 0.3 M NaCl and 35 mM imidazole. Alternatively, the cell lysate after centrifugation was treated in a water bath at 80°C for 30 min. After centrifugation at 12,000 g for 5 min, nearly pure isoamylase was obtained in the supernatant. The purity of the isoamylase was analyzing using 10% sodium dodecyl sulfate-polyacrilamide gel electrophoresis (SDS-PAGE). The protein bands were visualized by the staining of the Bio-Rad Coomassie Blue 250 staining kit. The protein concentration was measured with the Bio-Rad Bradford protein kit with bovine serum albumin as a reference.

The other recombinant proteins used for EFCs were prepared and purified as described elsewhere, including alpha-glucan phosphorylase (αGP), phosphoglucomutase (PGM), glucose 6-phosphate dehydrogenase (G6PDH) and diaphorase (DI) .

**Optimization of isoamylase reaction conditions**

To determine optimal pH, the reaction solution was mixed by 350 μl of 0.5% amylopectin solution, 100 μl of 0.2 M buffer (e.g., acetate buffers (pH 4.0-6.0) or phosphate buffers (pH 5.0-8.0)), and 50 μl of the enzyme solution (75 μg/ml). The mixture was incubated at 80℃ for 30 min. The reaction was stopped by using an ice bath. Ten μl of the enzymatic reaction mixture was mixed with 490 μl of distilled water and 500 μl of the bicinchonic acid (BCA) solution [^30^](#_ENREF_30). The tubes containing the reaction solutions were incubated at 75^o^C for 30 min. Concentrations of reducing ends were measured by the modified BCA assay with glucose as a reference [^30^](#_ENREF_30). To determine optimal temperature, the reaction mixtures in 40 mM acetate buffer (pH 5.5) were incubated at a temperature from 40 to 90℃ for 30 min. The reducing ends generated by IA were measured by the BCA assay. To determine optimal metal concentration, the reaction mixtures in 40 mM acetate buffer (pH 5.5) supplemented with 0.5 mM or 5 mM of CuCl_2_, FeCl_3_, ZnCl_2_, CaCl_2_, MgCl_2_, CoCl_2_, NiCl_2_, MnCl_2_ or EDTA were incubated at 80℃ for 30 min. The reducing ends generated by IA were measured by the BCA assay.

## **Isoamylase activity assay**

Isoamylase activity was measured in 500 μl of the reaction mixture containing 350 μl of 0.5% (wt/v) amylopectin solution, 100 μl of 0.2 M acetate buffer (pH 5.5) containing 2.5 mM of MgCl_2_, and 50 μl of the enzyme solution (75 μg/ml). The reaction mixtures were incubated at 80℃ for 30 min. The reducing ends generated by IA were measured by the modified BCA assay with glucose as a reference [^30^](#_ENREF_30). One international unit (IU) of isoamylase activity was defined as one micromole of reducing ends generated one min.

To determine the substrate specificity, the reaction mixture containing 350 μl of 0.5% (wt/v) solution containing amylopectin, amylose, pullulan, and maltodextrins, 100 μl of 0.2 M acetate buffer (pH 5.5) supplemented with 2.5 mM MgCl_2_, and 50 μl of the enzyme solution (75 μg/ml) was incubated at 80℃ for 30 min. The reducing ends generated by IA were measured by the BCA assay.

Alternatively, IA assay was measured by the increased blue value of glucan-iodine complexes as described elsewhere [^7^](#_ENREF_7). The reaction mixture contained 350 μl of 0.5% amylopectin solution, 100 μl of 0.2 M acetate buffer (pH5.5), 50 μl of the enzyme solution . The mixture was incubated at 80℃ for 30 min 300 rpm. A half ml of 0.005M I_2_-0.1M KI solution was added, followed by the addition of 10 ml of distilled water, and the mixed well. The increase in absorbance at 610 nm was measured.

## **Thermostability.**

Fifty μl of 75 μg/ml IA solution was diluted in 100 μl of 0.2 M acetate buffer (pH 5.5) containing 2.5 mM MgCl_2_ or CaCl_2_ or no divalent ions. The enzyme solutions were incubated at 70, 80 and 90 ℃ for different times. The remaining IA activities were measured as described previously.

**EFC preparation and measurement**

A cuvette enzymatic fuel cell was set up for testing as described previously [^2^](#_ENREF_2) with some modifications. Membrane electrode assembly including Nafion and cathode (1.8×2 cm; from Fuel Cell Earth Woburn, MA, USA) was adhered by epoxy glue to cover up the open window (0.5×1.5 cm) in a cuvette (1×1×4.5 cm). Oxygen in air acted as an electron acceptor. 1×1 cm carbon paper (Fuel Cell Earth Woburn, MA, USA) was anode. To test the effect of substrate on the performance of EFC, two types of anolyte solution were made by adding 0.012 (wt/v) % isoamylase-treated starch or 0.012% starch. The other enzymes in anolyte per ml were 7 U of αGP, 3 U of PGM, 1.5 U of G6PDH, 5.4 U of DI in a 50 mM HEPES buffer (pH 7.2) containing 0.3 M NaCl, 4 mM NAD^+^, 5 mM Mg^2+^, 0.25 mM Mn^2+^, and 1.7 mM, analogue antraquinone-2,6-disulfonate (AQDS) as an electron shuttle. All the electrochemical tests were performed on a 1000B Multi-Potentiostat (CH Instruments Inc., Austin, TX, USA) interfaced to a personal computer at room temperature (~20 ℃). Each test was repeated twice to ensure the reliability of data. For the linear sweep voltammetry (LSV), scanning was carried out at the rate of 5 mV s^-1^ after 10 min wait to measure EFC’s open circuit potential. For the cyclic voltammetry (CV) tests, the anolyte solution was aerated 20 min with nitrogen gas before testing to eliminate dissolved oxygen. Ag/AgCl electrode was used as a reference; platinum wire was applied as a counter electrode. The scanning rate was at 2 mV s^-1^.

# **Acknowledgments.**

This work could not be finished without the support of the Henan Agricultural University and Virginia Tech. This work was partially supported by the special fund for agro-scientific research in the public interest from the Ministry of Agriculture, China (No. 201503134). In addition, funding to YPZ for this work was provided in part, by NSF SBIR II award (IIP-1353266) and by the Virginia Agricultural Experiment Station and the Hatch Program of the National Institute of Food and Agriculture, U.S. Department of Agriculture.

# **References**

1. Calabrese Barton, S., Gallaway, J. & Atanassov, P. Enzymatic biofuel bells for implantable and microscale devices. *Chem. Rev.* **104**, 4867-4886 (2004).

2. Zhu, Z.-G., Kin Tam, T., Sun, F., You, C. & Zhang, Y.-H.P. A high-energy-density sugar biobattery based on a synthetic enzymatic pathway. *Nat. Commun.* **5**, 3026 (2014).

3. Cracknell, J.A., Vincent, K.A. & Armstrong, F.A. Enzymes as working or inspirational electrocatalysts for fuel cells and electrolysis. *Chem. Rev.* **108**, 2439-2461 (2008).

4. Moehlenbrock, M. & Minteer, S. Extended lifetime biofuel cells. *Chem. Soc. Rev.* **37**, 1188-1196 (2008).

5. Sakai, H. et al. A high-power glucose/oxygen biofuel cell operating under quiescent conditions. *Energy Environ. Sci.* **2**, 133-138 (2009).

6. Park, H.-S. et al. TreX from *Sulfolobus solfataricus* ATCC 35092 Displays Isoamylase and 4-α-Glucanotransferase Activities. *Biosci. Biotechnol. Biochem.* **71**, 1348-1352 (2007).

7. Fang, T.-Y., Tseng, W.-C., Yu, C.-J. & Shih, T.-Y. Characterization of the thermophilic isoamylase from the thermophilic archaeon *Sulfolobus solfataricus* ATCC 35092. *J. Mol. Catal. B: Enzymatic* **33**, 99-107 (2005).

8. Li, Y., Niu, D., Zhang, L., Wang, Z. & Shi, G. Purification, characterization and cloning of a thermotolerant isoamylase produced from *Bacillus* sp. CICIM 304. *J. Ind. Microbiol. Biotechnol.* **40**, 437-446 (2013).

9. Zhang, Y.-H.P. Renewable carbohydrates are a potential high density hydrogen carrier. *Int. J. Hydrogen Energy* **35**, 10334-10342 (2010).

10. Zhang, Y.-H.P. Next generation biorefineries will solve the food, biofuels, and environmental trilemma in the energy-food-water nexus. *Energy Sci. Eng.* **1**, 27-41 (2013).

11. Wang, Y. & Zhang, Y.-H.P. Cell-free protein synthesis energized by slowly-metabolized maltodextrin. *BMC Biotechnol.* **9**, 58 (2009).

12. Caschera, F. & Noireaux, V. A cost-effective polyphosphate-based metabolism fuels an all *E. coli* cell-free expression system. *Metab. Eng.* **27**, 29-37 (2015).

13. Fessner, W.-D. Systems Biocatalysis: Development and engineering of cell-free “artificial metabolisms” for preparative multi-enzymatic synthesis. *New Biotechnol.* (2015).

14. Bornscheuer, U.T. et al. Engineering the third wave of biocatalysis. *Nature* **485**, 185-194 (2012).

15. Zhang, Y.-H.P. Production of biofuels and biochemicals by in vitro synthetic biosystems: opportunities and challenges. *Biotechnol. Adv.*, DOI: 10.1016/j.biotechadv.2014.1010.1009 (2015).

16. Kawarabayasi, Y. et al. Complete Genome Sequence of an Aerobic Thermoacidophilic Crenarchaeon, Sulfolobus tokodaii strain7. *DNA Research* **8**, 123-140 (2001).

17. Li, Y., Zhang, L., Ding, Z. & Shi, G. Constitutive expression of a novel isoamylase from *Bacillus lentus* in *Pichia pastoris* for starch processing. *Proc. Biochem.* **48**, 1303-1310 (2013).

18. Dauvillée, D. et al. Role of the *Escherichia coli* glgX Gene in Glycogen Metabolism. *J. Bacteriol.* **187**, 1465-1473 (2005).

19. Maruta, K., Kubota, M., Fukuda, S. & Kurimoto, M. Cloning and nucleotide sequence of a gene encoding a glycogen debranching enzyme in the trehalose operon from *Arthrobacter* sp. Q36. *Biochim. Biophys. Acta - Prot. Struct. Mol. Enzymol.* **1476**, 377-381 (2000).

20. Abe, J.-i., Ushijima, C. & Hizukuri, S. Expression of the Isoamylase Gene of *Flavobacterium odoratum* KU in Escherichia coliand Identification of Essential Residues of the Enzyme by Site-Directed Mutagenesis. *Appl. Environ. Microbiol.* **65**, 4163-4170 (1999).

21. Katsuya, Y., Mezaki, Y., Kubota, M. & Matsuura, Y. Three-dimensional structure of *Pseudomonas* isoamylase at 2.2 Å resolution. *J. Mol. Biol.* **281**, 885-897 (1998).

22. Lim, W.J. et al. Cloning and Characterization of an Intracellular Isoamylase Gene from *Pectobacterium chrysanthemi* PY35. *Biochem. Biophys. Res. Commun.* **287**, 348-354 (2001).

23. Ara, K., Saeki, K. & Ito, S. Purification and characterization of an alkaline isoamylase from an alkalophilic strain of *Bacillus*. *J. Gen. Microbiol.* **139**, 781-786 (1993).

24. Takashima, Y. et al. Differential Chain-Length Specificities of Two Isoamylase-Type Starch-Debranching Enzymes from Developing Seeds of Kidney Bean. *Biosci. Biotechnol. Biochem.* **71**, 2308-2312 (2007).

25. You, C., Zhang, X.-Z. & Zhang, Y.-H.P. Simple Cloning via direct transformation of PCR product (DNA multimer) to *Escherichia coli* and *Bacillus subtilis*. *Appl. Environ. Microbiol.* **78**, 1593-1595 (2012).

26. Myung, S. & Zhang, Y.-H.P. Non-complexed four cascade enzyme mixture: simple purification and synergetic co-stabilization. *PLos One* **8**, e61500 (2013).

27. Ninh, P.H., Honda, K., Sakai, T., Okano, K. & Ohtake, H. Assembly and Multiple Gene Expression of Thermophilic Enzymes in *Escherichia coli* for In Vitro Metabolic Engineering. *Biotechnol. Bioeng.* **112**, 189-196 (2015).

28. Zhu, Z.G., Wang, Y.R., Minteer, S. & Zhang, Y.-H.P. Maltodextrin-powered enzymatic fuel cell through a non-natural enzymatic pathway. *J. Power Sources* **196**, 7505-7509 (2011).

29. You, C. et al. Enzymatic transformation of nonfood biomass to starch. *Proc. Nat. Acad. Sci. USA* **110**, 7182-7187 (2013).

30. Zhang, Y.-H.P. & Lynd, L.R. Determination of the number-average degree of polymerization of cellodextrins and cellulose with application to enzymatic hydrolysis. *Biomacromolecules* **6**, 1510-1515 (2005).

31. Krohn, B.M., Barry, G.F. & Kishore, G.M. An isoamylase with neutral pH optimum from a *Flavobacterium* species: cloning, characterization and expression of the iam gene. *Molec. Gen. Genet.* **254**, 469-478 (1997).

32. Yokobayashi, K., Misaki, A. & Harada, T. Purification and properties of *Pseudomonas* isoamylase. *Biochim. Biophys. Acta.* **212**, 458-469 (1970).

33. Fujita, N. et al. Purification, characterization, and cDNA structure of isoamylase from developing endosperm of rice. *Planta* **208**, 283-293 (1999).

34. Hussain, H. et al. Three Isoforms of Isoamylase Contribute Different Catalytic Properties for the Debranching of Potato Glucans. *Plant Cell* **15**, 133-149 (2003).

**Contributions**

PZ, KC and HC wrote the main manuscript text. KC conducted experiments pertaining to isoamylase production, purification and characterization. FZ and FS conducted experiments pertaining to sugar biobatteries and prepared figure 6. All authors reviewed the manuscript.

# **Additional Information -- Competing financial interests**

KC, HC and PZ declare competing financial interests in the form of a provisional patent for this newly-discovered hyperthermophilic isoamylase, filed by Henan Agricultural University.

# **Figure Legends**

**Figure 1**. The scheme of amylopectin hydrolysis catalyzed by isoamylase (IA) for the generation of linear amylodextrin (a) and of an air-breathing biobattery powered by amylopectin or starch (b). The enzymes used are α-glucan (starch) phosphorylase (αGP), phosphoglucomutase (PGM), glucose-6-phosphate dehydrogenase (G6PDH), and diaphorase (DI).

**Figure 2**. Comparison of the conserved amino acid sequences in the active sites of isoamylases. Isoamylase sources (gene ID) are *S. tokodaii* (1458890), *S. solfataricus* (384432549), *S. acidocaldaricus* (568309602), *E. coli F11* (190908135), *Arthrobacter* sp. (7648481), *E. chrysanthemi* (22074054), *P. amylodermosa* (151294), *F. odoratum* (5672639), *B. lentus* (493116169), and *P. vulgaris* (kidney bean) (139867062).

**Figure 3**. SDS-PAGE analysis of isoamylase expression and purification in *E. coli* BL21 (DE3) and Rosetta (DE3) (a). Lanes: M, markers; B, BL21 host; R, Rosetta host; S, the supernatant of the cell lysate of *E. coli* Rosetta; T, the cell lysate of *E. coli* Rosetta; P: pellets of the cell lysate of *E. coli* Rosetta; HT, the supernatant of the heat-treated cell lysate of *E. coli* Rosetta; and His, the purified isoamylase by using Ni-charged resins. Effect of pH on the isoamylase activity (b). Buffer concentration was 40 mM and 0.5 mM MgCl_2_: acetate buffer (pH 4-6) and phosphate buffer (pH 5-8). Data represent the mean ± S.D. from triplicate experiments. Effect of temperature on the isoamylase activities (c). Reaction conditions were 40 mM acetate buffer (pH 5.5) containing 0.5 mM MgCl_2_. Data represent the mean ± S.D. from triplicate experiments.

**Figure 4.** Photos of iodine dyed amylopectin and isoamylase treated amylopectin (a) and light absorption spectrum of the iodine-stained amylopectin compared with isoamylase treated amylopectin. Reaction conditions were 0.75% amylopectin in 40 mM acetate buffer (pH 5.5) containing 0.5 mM MgCl_2_ and 7.5 μg/ml isoamylase incubated at 80℃ for 30 min. The stained samples were diluted by a factor of 10 in water.

**Figure 5**. The stability profile of isoamylase in the presence of 0.5 mM MgCl_2_, 0.5 mM CaCl_2_  or the absence of bivalent metal ions at 90℃. The buffer was 40 mM acetate buffer (pH 5.5) containing 7.5 μg/ml isoamylase.

**Figure 6**. Representative polarization and power curves for biobatteries powered by isoamylase-treated starch (a) and non-treated starch (b). Cyclic voltammetry curves of biobatteries powered by isoamylase-treated starch and non-treated starch (c). Photo of a cuvette-based air-breathing biobattery.

# **Table 1.**

Comparison of basic properties of characterized isoamylases.

| Organism | Gene ID | Protein size (AA) | Opt. Temp & pH | Sp act. IU/mg (U/mg*) | Half life time (condition) | Ref. |
| --- | --- | --- | --- | --- | --- | --- |
| *Sulfolobus tokodaii* | 24473558 | 716 | 85 ^o^C, pH 5.5 | 6.4 ( 1759*) | 3.5 h (90^o^C, + Mg^2+^) | This Study |
| *Sulfolobus solfataricus* | 15896971 | 718 | 75 ^o^C, pH 5.0 | 16.7 (4600*) | 2 h (85^o^C) | [^7^](#_ENREF_7) |
| Bacillus lentus | 493116169 | 886 | 70^o^C, pH 5.5-8.5 | 53 (6,535*) | < 1 h, 80 ^o^C |  |
| *Flavobacterium*  odoratum | 5672639 | 762 | 37 ^o^C, pH 6.0-7.0 | 172 | NA | [^31^](#_ENREF_31) |
| Phaseolus vulgaris (kidney bean) | 139867062 | 783 | 30 ^o^C, pH 6.0 | 3.51 | NA | [^24^](#_ENREF_24) |
| *Pseudomonas amyloderamosa* | 151294 | 771 | 52 ^o^C, pH 3-4 | 59 | NA | [^32^](#_ENREF_32) |
| *Oryza sativa (rice)* | 3252793 | 733 | 30 ^o^C, pH 6.5-7 | 51.2 | NA | [^33^](#_ENREF_33) |
| *Pectobacterium chrysanthemi* PY35 | 22074054 | 657 | 40 ^o^C, pH 7.0 | NA | NA | [^22^](#_ENREF_22) |
| *Solanum tuberosum (potato)* | 568214804 | 793 | 30 ^o^C, pH 6 | 0.1 | NA | [^34^](#_ENREF_34) |

* Not international unit. Unit was measured based on the iodine-stain method.
